# The Evaluation of Eazyplex^®^ SuperBug CRE Assay Usefulness for the Detection of ESBLs and Carbapenemases Genes Directly from Urine Samples and Positive Blood Cultures

**DOI:** 10.3390/antibiotics11020138

**Published:** 2022-01-21

**Authors:** Alicja Sękowska, Tomasz Bogiel

**Affiliations:** Department of Microbiology, Ludwik Rydygier Collegium Medicum in Bydgoszcz, Nicolaus Copernicus University in Torun, 9 M. Sklodowska-Curie Street, 85-094 Bydgoszcz, Poland; t.bogiel@cm.umk.pl

**Keywords:** carbapenemases, Eazyplex^®^ SuperBug CRE assay, extended-spectrum beta-lactamases, gram-negative rods, LAMP method

## Abstract

Increasing antimicrobial resistance of Gram-negative rods is an important diagnostic, clinical and epidemiological problem of modern medicine. Therefore, it is important to detect multi-drug resistant strains as early on as possible. This study aimed to evaluate Eazyplex^®^ SuperBug CRE assay usefulness for beta-lactamase gene detection among Gram-negative rods, directly from urine samples and positive blood cultures. The Eazyplex^®^ SuperBug CRE assay is based on a loop-mediated isothermal amplification of genetic material and allows for the detection of a selection of genes encoding carbapenemases, KPC, NDM, VIM, OXA-48, OXA-181 and extended-spectrum beta-lactamases from the CTX-M-1 and CTX-M-9 groups. A total of 120 clinical specimens were included in the study. The test gave valid results for 58 (96.7%) urine samples and 57 (95.0%) positive blood cultures. ESBL and/or carbapenemase enzymes genes were detected in 56 (93.3%) urine and 55 (91.7%) blood samples, respectively. The Eazyplex^®^ SuperBug CRE assay can be used for a rapid detection of the genes encoding the most important resistance mechanisms to beta-lactams in Gram-negative rods also without the necessity of bacterial culture.

## 1. Introduction

Bacteria of Enterobacterales, especially multi-drug resistant isolates, are one of the most important causes of nosocomial infections. Their significance as a global threat can be explained in several ways; an easy acquisition of antibiotic resistance genes and the capability of these strains to survive in the hospital environment and antimicrobial pressure/bacterial evolution as an answer to common application of a broad-spectrum antibiotic therapy. The problem of multi-drug resistance of Gram-negative rods is mostly associated with the horizontal gene transfer and possible synthesis of several beta-lactamases from the same or different groups of enzymes (e.g., ESBLs-extended spectrum beta-lactamases, carbapenemases). It mainly includes enzymes with a wide range of activity (cephalosporinases or carbapenemases) whose incidences have risen enormously in the last two decades [1,2,3]. These enzymes have evolved significantly since their discovery and several variants of each enzyme can be currently identified within one enzyme family.

Numerous phenotypic and genotypic methods used in microbiological laboratories allow for the detection of ESBLs and carbapenemase-producing strains. Phenotypic methods always require the culture of the strain, which extends the time needed to obtain a result. On the other hand, these methods are simple to perform and relatively inexpensive, but usually do not allow the identification of a specific enzyme type or variant [4]. An introduction of molecular biology-based methods for routine microbiological diagnostics allows for a simultaneous detection of different antimicrobial resistance genes, also within one strain. Additionally, it enables the identification of a specific enzyme within the bacteria group or family and also directly from a clinical sample or a specimen pre-culture [1,2].

The evaluated Eazyplex^®^ SuperBug CRE assay is based on the Loop-mediated isothermal amplification (LAMP) technique and can be used for the detection of ESBL enzyme genes from CTX-M-1 and CTX-M-9 groups, as well as VIM- (1–37), NDM- (1–7), KPC and OXA-48-like (-48, -162, -204, -244) carbapenemase gene variants.

The aim of this study was to evaluate the usefulness of eazyplex^®^ SuperBug CRE assay for the detection of the genes encoding for the most important beta-lactamases among Gram-negative rods, directly from urine samples and positive blood cultures. The results of the evaluated assay were also compared with the results of conventional methods applied (phenotypic tests for beta-lactamases detection), the results of the standard PCR, and evaluated as an alternative tool for rapid and reliable antimicrobial resistance detection in a large, multidisciplinary university hospital.

## 2. Results

Of 60 urine samples tested, 40 *K. pneumoniae* strains, 13 strains of *Escherichia coli*, three of *K. oxytoca*, three *Enterobacter cloacae* and one *Klebsiella variicola* strain were recovered. All the strains were isolated in monoculture at a titer of ≥10^4^ CFU/mL. The eazyplex^®^ SuperBug CRE assay performed for 58 (96.7%) samples gave valid results. In 51 samples ESBLs genes were detected exclusively, and an additional five samples-ESBLs and carbapenemases genes were present simultaneously (Table 1).

The detection time ranged from 4:30 min to 11:45 min for the ESBL enzyme genes, and from 8:15 min to 15:15 min for the carbapenemases genes.

For 58 strains derived from urine samples, double-disc synergy test results revealed a characteristic enlargement of the growth inhibition zones between the discs from the side of the beta-lactamase containing disc, which confirmed the presence of ESBLs. For two *K. pneumoniae* strains the results were not interpretable due to the lack of any inhibition zone around the discs applied for ESBLs detection. For five carbapenem-resistant strains with positive results via the Carba NP test and EDTA-supplemented disc method, positive results were obtained in the Eazyplex^®^ SuperBug CRE assay. With the application of standard PCR for the chosen 44 (73.3%) strains (40 *K. pneumoniae*, 3 *K. oxytoca* and 1 *K. variicola*), *bla*_CTX-M_ genes presence was confirmed amongst 39 *K. pneumoniae* strains (including negative and non-valid results of Eazyplex^®^ SuperBug CRE assay), while *bla*_TEM_ and *bla*_SHV_ were detected amongst 34 and 26 isolates, respectively (Table 2).

For the *K. variicola* strain, the consent results were obtained with the application of the Eazyplex^®^ SuperBug CRE assay and standard PCR. The results for two *K. oxytoca* strains were concordant for the evaluated assay and standard PCR, while one non-valid Eazyplex^®^ SuperBug CRE assay result accompanied with the presence of *bla*_CTX-M_ and *bla*_TEM_ genes in a standard PCR. Comparing the results obtained with the evaluated test based on LAMP method and standard PCR methods, a categorical agreement was obtained for 40 out of 44 ESBL-positive strains and for all three carbapenemase-producing isolates. Comparing the results obtained with the evaluated test and phenotypic methods, a categorical compliance was obtained for 56 out of 60 ESBL-positive strains and for all five carbapenemase producing isolates derived from urine samples.

Of 60 positive blood cultures tested, 46 *K. pneumoniae* strains, three strains of each: *K. oxytoca*, *E. cloacae* and *E. coli*, two strains of *S. marcescens* and *Citrobacter freundii*, and one *Proteus mirabilis* strain were derived. Fifty eight samples (96.7%) gave the strains isolated in monoculture, while from two samples, *K. pneumoniae* and *Enterococcus faecalis,* strains were grown simultaneously. For 59 strains derived from positive blood cultures, ESBLs presence was confirmed with the application of a double disc synergy test.

The Eazyplex^®^ SuperBug CRE assay gave valid results for fifty seven (95.0%) samples of pre-cultured blood; In 50 samples only ESBL enzyme genes were detected exclusively, and an additional four samples-ESBLs and carbapenemases genes were present simultaneously (Table 3).

The detection time for ESBL genes ranged from 4:30 min to 10:15 min, while for carbapenemases genes times ranged from 4:45 min to 11:45 min. Comparing the results obtained for 50 (83.3%) of the chosen blood-derived strains (45 *K. pneumoniae*, 3 *K. oxytoca* and 2 *S. marcescens*) by the application of the evaluated test and standard PCR, a categorical agreement was obtained for 48 out of 50 ESBL-positive strains and for all the carbapenemase-producing strains. Comparing the results obtained by the assay based on the LAMP technique and standard PCR results, a categorical compliance was obtained for 54 out of 60 ESBL-positive strains and for all 5 carbapenemase producing isolates (Table 4).

The presence of *bla*_CTX-M_ genes in 44 *K. pneumoniae* strains, *bla*_TEM_ in 31 and *bla*_SHV_ in 25 was confirmed by standard PCR (including negative and non-valid results of the Eazyplex^®^ SuperBug CRE assay) (Table 4).

One *K. pneumoniae* strain (negative in the evaluated assay) revealed the *bla*_TEM_ gene, while for the samples with invalid results in the Eazyplex^®^ SuperBug the CRE assay-*bla*_CTX-M_ gene was confirmed with the application of a standard PCR. The results obtained by the evaluated assay and standard PCR methods were also consistent for three *K. oxytoca* strains. For one *S. marcescens* strain, the results obtained with the Eazyplex^®^ SuperBug CRE assay were consistent with the standard PCR method, while for the second *S. marcescens* strain (negative in Eazyplex^®^ SuperBug CRE assay) *bla*_CTX-M_ and *bla*_TEM_ genes were detected. For *E. coli* and *P. mirabilis* strains that were negative in Eazyplex^®^ SuperBug CRE assay, ESBLs production was confirmed by the double disc synergy test.

Five strains, isolated from positive blood cultures, suspected of producing carbapenemases in the Carba NP test, were also positive with the application of the Eazyplex^®^ SuperBug CRE assay. Among four *K. pneumoniae* strains, production of metallo-beta-lactamases was confirmed by means of EDTA-supplemented discs, and the NDM-1 carbapenemase genes by standard PCR. For one *K. pneumoniae* strain, the synthesis of KPC was confirmed by the test with boronic acid. In one *K. pneumoniae* strain producing KPC-type carbapenemases, no growth inhibition zones were observed around the discs when the phenotypic methods for ESBL detection were applied. Neither the presence of the genes encoding ESBL from the CTX-M1 and CTX-M9 group was confirmed in the Eazyplex^®^ SuperBug CRE assay, nor were *bla*_CTX-M_, *bla*_TEM_ or *bla*_SHV_ genes detected by standard PCR.

## 3. Discussion

In recent years, the frequency of multi-drug-resistant bacterial strains isolation has increased significantly [5,6,7]. This is mainly due to bacterial spread in hospital environment and unreasonable antibiotic therapy. Antimicrobial pressure on bacterial strains causes the emergence of new mechanisms of antibiotic resistance. Therefore, it is crucial to obtain reliable results of the presence of antimicrobial resistance in the shortest time possible.

It is commonly known that the application of a test which simultaneously detects ESBL and carbapenemase enzymes, or their genes, significantly shortens the time of standard microbiological diagnostics. In addition, results of the phenotypic test performed for carbapenemase-producing strains are often ambiguous or difficult to interpret. For example, in the results of a double-disc synergy test the enlargement of the growth inhibition zones, characteristic for ESBL-positive strains, sometimes does not have a typical shape or does not appear at all. Thus, it requires an application of further methods which sometimes significantly extends the time to give a final result.

The Eazyplex^®^ SuperBug CRE assay is based on isothermal amplification of a genetic material and detects the genes for the following enzymes: KPC, NDM, VIM, OXA-48, CTX-M-1, CTX-M-9 and OXA-181. In the available literature, the first studies on Eazyplex^®^ SuperBug CRE assay application appeared in 2015 [1,8]. The mentioned studies were performed on 94 and 450 carbapenemase positive Gram-negative rods strains, respectively. The results of the mentioned research indicated a high sensitivity and specificity of Eazyplex^®^ SuperBug CRE assay in the detection of resistance mechanisms genes in Gram-negative rods [1,2,8]. The study published by Hinić et al. [9], also in 2015, described the use of the Eazyplex test for the detection of genes of ESBL-positive Gram-negative rods directly in 50 urine samples. In 30 (60.0%) of them the presence of ESBL was confirmed. Thus, the overall sensitivity of the method reached from 95.2% to 100% with a specificity of 97.9%.

In the present study, ESBL and/or carbapenemase enzymes were detected in more than 93% of urine samples. The median detection time of ESBL enzyme genes from urine samples was 7 min 45 s, while it was 9:45 min for carbapenemases genes. For two urine samples with negative results in the Eazyplex^®^ SuperBug CRE assay, the *bla*_TEM_ gene presence was confirmed by a standard PCR. It is noteworthy that this gene was not detected by the evaluated assay. In the second strain, three different genes encoding ESBL enzymes were detected by the applied confirmatory standard PCR. The false-negative result of the Eazyplex^®^ SuperBug CRE assay for the second strain might have resulted from the low number of gene copies below the assay detection limit. However, the manufacturer assures us that the evaluated test can efficiently detect a small number of gene copies (100% sensitivity).

Recently, Fiori et al. [10] evaluated the usefulness of Eazyplex^®^ SuperBug CRE assay for ESBL and/or carbapenemases genes detection directly in positive blood cultures. The mentioned authors detected the presence of CTX-M and/or KPC and/or VIM-like enzymes genes in 151 of the pre-cultured blood samples among 321 episodes of bloodstream infections. The results obtained by this method allowed for the reduction of time to effective antibiotic therapy introduction in patients with *E. coli* or *K. pneumoniae* bacteraemia. The cited authors also highlight the proposed algorithm for combination of a mass spectrometry identification, directly from a blood sample, and the detection of a resistance mechanism with Eazyplex^®^ SuperBug CRE assay, which significantly reduces diagnostic procedures and the time required to get the final results.

In our study, the median detection time of ESBL enzyme genes from the positive blood cultures was 6 min and for carbapenemases genes it was 7 min 30 s when compared to an overnight incubation of the phenotypic test for a particular antimicrobial resistance mechanism detection. Also, results from Rödel et al. [11] indicate a possible reduction of time and the rationalization of antibiotic therapy in the case of patients with sepsis using the Eazyplex test. However, the mentioned authors used an Eazyplex^®^ MRSA test to detect *mecA* and *mecC* genes among *Staphylococcus aureus* and *Staphylococcus epidermidis* strains.

In turn, Bach et al. [12] assessed the usefulness of the LAMP method-based assay in the identification of selected bacterial species and genes encoding ESBL enzymes from the CTX-M1 and CTX-M9 groups from positive blood cultures. The study included 449 positive blood cultures. In the aforementioned research, with the application of the Eazyplex^®^ BloodScreen GN assay, 100% sensitivity and specificity were obtained for *K. oxytoca* and *E. coli* strains, while 95.7% sensitivity and 100% specificity was obtained for *K. pneumoniae* isolates infections. When assessing the detection of genes encoding CTX-M enzymes, they also obtained 100% sensitivity and specificity.

Slightly over 4% of the tests performed in the present work were not valid. This can be explained by several reasons: sample overload, specimen composition, consistency, the presence of some inhibitory substances in urine or blood samples (patients from whom the samples were collected from often take a number of drugs), the presence of more than one strain in a single sample, the presence of several genes encoding for several resistance mechanisms simultaneously, or particular genes mutations. It may lead to the necessity of repeating the whole procedure, which additionally increases the cost and time of the investigation or leads to the underestimation and oversight of resistance mechanism presence if not consequently repeated.

It is worthy of note that the current the number of recognized beta-lactamases is estimated at around 200 or more. In the double disc synergy tests, it is not possible to identify a specific ESBL family, or whether the strain produces one or more ESBL-like enzymes. In a standard PCR method, it is possible to detect many different ESBL enzymes genes, but this requires a more complex approach, usually a longer time, and often several PCR reactions (or a multiplex approach) to detect specific genes, which also affects the costs. Moreover, whether a strain produces more than one type of ESBLs is of epidemiological significance only and does not clinically affect the antibiotic therapy approach. Hence, the available commercial tests (including the Eazyplex^®^ SuperBug CRE assay) are usually designed to detect the most common antibiotic resistance mechanisms among isolated strains, the selected genes encoding for ESBL and/or carbapenemases.

When comparing the methods used in the study, it should be noted that the phenotypic methods (the double disc synergy test and the Carba NP test) are simple to perform, relatively inexpensive (approximately $2 and $1.5 for one strain, respectively), but require earlier culture (which is related to time delay-16–20 h) and do not allow for the identification of a specific ESBL enzyme or carbapenemase type. On the other hand, the standard PCR method for several genes is more expensive (about $10), requires specialized equipment and laboratories as well as carrying out a multiplexed or multiple separate reactions with different primers (which is associated with a time delay of several hours). The LAMP method, on the other hand, is quite expensive (around $50) but also very fast (less than 20 min). In addition, this method allows for the identification of a specific enzyme type, which is very important in epidemiological studies.

In the present study a great advantage of the Eazyplex^®^ SuperBug CRE assay to obtain a result directly from a clinical sample was confirmed. A short duration of the test and a small sample volume needed to perform are also very important. However, the test has some limitations. It detects only ESBL enzymes from the CTX-M-1 and M-9 groups. On the other hand, CTX-M enzymes are the most common ESBL enzymes in *K. pneumoniae*, and the strains with this resistance mechanism are isolated with the highest frequency, not only in Poland, but also worldwide [13,14,15,16,17]. Moreover, the evaluated assay does not detect all beta-lactamases genes, like ampC-like enzymes. However, the dissemination of ampC-positive *Klebsiella* spp. isolates, at least in our department, does not exceed 1% (data not shown) and thus it was not the issue of the present work. The study also did not compare all the tested strains using the Eazyplex^®^ SuperBug CRE assay and standard PCR, which is a limitation of the study, but it was not possible in this research protocol and was not the key goal of the study.

It is worthy of note that the Eazyplex^®^ SuperBug CRE assay detects only some chosen enzymes genes also among carbapenemases. Interestingly, the strains of Gram-negative rods, derived from the patients in our hospital, that express beta-lactams resistance mainly synthesize class B carbapenemases, mostly the NDM- or VIM-type. Detection of the genes for both mentioned carbapenemases is available in the evaluated assay. Of note, not all types of carbapenemases have been detected among the strains of Gram-negative rods in our hospital so far. Since the first detection of a carbapenemase producing strain in our laboratory, only one KPC-positive and one OXA-48 positive strain have been identified (data not shown). It confirms that the strains expressing this particular resistance mechanism are very rare in our department. Nevertheless, the study was limited to the assessment of the usefulness of the Eazyplex^®^ SuperBug CRE assay only for strains of the Enterobacterales order. On the other hand, the possibility of detecting selected genes in the above assay applies mainly to Enterobacterales rods, and to a lesser extent to bacteria belonging to *Pseudomonas* spp.

The Eazyplex^®^ SuperBug CRE assay is a relatively expensive tool for its use in a routine diagnostic directly for clinical specimens. This approach would significantly increase the cost of a standard microbiological investigation. However, its application might be reasonably taken into account in the diagnostic of some particular cases of infections, such as earlier colonization of the patients with ESBL- or carbapenemase-producing strains, confirmed contact with the infected person, or the presence of the local epidemic outbreak. It seems that in such situations the benefits of using an expensive test are favourable for obtaining the result in a shorter time. Moreover, the possibility of introducing a pre-emptive treatment, while also taking into account the phenotype of the strain, seems to be a very reasonable approach.

## 4. Materials and Methods

The study included 120 clinical specimens: 60 urine samples and 60 positive blood cultures, in which Gram-negative strains producing beta-lactamases with a broad range of activity: ESBLs and/or carbapenemases were identified. Clinical specimens for the present research were mostly selected based on local epidemiological data. Urine samples are the most common type of clinical material sent for testing from hospital patients, while blood is the main material in the microbiological diagnosis of systemic infections. Each sample included into the study was collected from a specific patient. All the samples included into the study were obtained through a routine diagnostic and clinical microbiology laboratory practice. The identification of the strains was performed by mass spectrometry using the MALDI Biotyper system (Bruker, Karlsruhe, Germany). In the analyzed study period, *K. pneumoniae* strains were the most frequently isolated species from infections in patients in our hospital. Antimicrobial susceptibility testing was determined on a BD Phoenix™ M50 instrument (Becton-Dickinson, Franklin Lakes, NJ, USA) using NMIC-402 panels, performed according to the manufacturer’s instructions. The expression of ESBL-type enzymes was assessed simultaneously by the double disc synergy test [18], while carbapenemases activity was assessed by the Carba NP test [19]. Phenotypic tests with the application of boronic acid and EDTA-supplemented discs, as specific carbapenemases inhibitors, were also applied, as recommended by EUCAST documents. For all of the original samples stored from which Gram-negative beta-lactamase-producing rods were cultured, the Eazyplex^®^ SuperBug CRE assay (Amplex Diagnostics, Gieβen, Germany) was performed on a Genie II device (OptiGene, Gieβen, Germany) according to the manufacturer’s instructions [20] (see also Supplementary Material).

Additionally, the chosen 44 strains (all *Klebsiella* spp.) isolated from urine samples and the 50 derived from pre-cultured blood (48 *Klebsiella* spp. and 2 *Serratia marcescens* isolates) were cultured on LB Broth (Biocorp, Issoire, France) to confirm the selected genes presence at the further steps. DNA was extracted from the strains recovered from them (applying an Extractme DNA Bacteria Kit, Blirt, Gdańsk, pomeranian, Poland) and the following genes were detected by standard PCR: *bla*_CTX-M_, *bla*_TEM_, *bla*_SHV_, *bla*_VIM_ and *bla*_NDM-1_ in separate reactions, according to the methodology of the previous studies [2,21,22].

## 5. Conclusions

The Eazyplex^®^ SuperBug CRE assay can be a useful tool for a rapid and reliable identification of resistance mechanism genes in Gram-negative rods, and also directly from urine and pre-cultured blood samples.

## Figures and Tables

**Table 1:** Beta-lactamases genes detected in Eazyplex^®^ SuperBug CRE assay and PCR performed directly on urine samples (*n* = 60) and the enzymes activity detected with the application of a particular phenotypic method.

Species Recovered from the Samples	Beta-Lactamases Genes Detected by Eazyplex^®^	No. of Isolates	Time of Particular Genes Detection by Eazyplex^®^ (min:s)	Double Disc Synergy Test Result	Carba NP Test Result
*K. pneumoniae* (*n* = 40)	CTX-M-1	32	4:30–10:15	(+)	N/A
	CTX-M-1, CTX-M-9	2	5:30–8:30	(+)	N/A
	CTX-M-1, NDM	3	5:15–15:15	(+)	(+)
	Negative result	2	-	UI	N/A
	Invalid test	1	-	(+)	N/A
*E. coli* (*n* = 13)	CTX-M-1	9	6:30–11:45	(+)	N/A
	CTX-M-9	3	7:00–8:45	(+)	N/A
	CTX-M1, VIM	1	5:30, 9:15	(+)	(+)
*E. cloacae* (*n* = 3)	CTX-M-1	2	6:45–7:00	(+)	N/A
	CTX-M-1, VIM	1	6:00, 8:15	(+)	(+)
*K. oxytoca* (*n* = 3)	CTX-M-1	1	9:45	(+)	N/A
	CTX-M-9	1	7:00	(+)	N/A
	Invalid test	1	-	(+)	N/A
*K. variicola* (*n* = 1)	CTX-M1	1	7:30	(+)	N/A

UI—uninterpretable-lack of any growth inhibition zone in the applied double disc synergy test (criteria described in the text); (+)—positive results in the applied method, N/A—not applicable.

**Table 2:** Comparison of the results obtained using Eazyplex^®^ SuperBug CRE assay and standard PCR for the chosen urine-derived strains (*n* = 44).

Species Recovered from the Samples	Eazyplex^®^ SuperBug CRE Assay	No. of Isolates	Standard PCR	No. of Isolates
*K. pneumoniae* (*n* = 40)	CTX-M-1 CTX-M-1, CTX-M-9 CXT-M1, CTX-M-9 CTX-M-1, NDM Negative result Negative result Invalid test	32 1 1 3 1 1 1	CTX-M, TEM, SHV CTX-M, TEM CTX-M CTX-M, TEM, SHV CTX-M, TEM CTX-M, NDM CTX-M, TEM, SHV TEM CTX-M, TEM, SHV	23 6 3 1 1 3 1 1 1
*K. oxytoca* (*n* = 3)	CTX-M-1 CTX-M-9 Invalid test	1 1 1	CTX-M CTX-M CTX-M, TEM	1 1 1
*K. variicola* (*n* = 1)	CTX-M-1	1	CTX-M	1

**Table 3:** Beta-lactamases genes detected with Eazyplex^®^ SuperBug CRE assay and standard PCR while performed on positive blood cultures (*n* = 60) and the enzymes activity detected with the application of a particular phenotypic method.

Species Recovered from the Samples	Beta-Lactamases Genes Detected by Eazyplex^®^	No. of Isolates	Time of Particular Genes Detection by Eazyplex^®^ (min:s)	Double Disc Synergy Test Result	Carba NP Test Result
*K. pneumoniae* (*n* = 46)	CTX-M-1	39	4:30–10:15	(+)	N/A
	CTX-M-1, CTX-M-9, NDM	1	4:45–5:15	(+)	(+)
	CTX-M-1, NDM	3	4:45–11:45	(+)	(+)
	KPC	1	9:15	UI	(+)
	Negative result	1	-	(+)	N/A
	Invalid test	1	-	(+)	N/A
*E. cloacae* (*n* = 3)	CTX-M-1	3	6:15–7:30	(+)	N/A
*E. coli* (*n* = 3)	CTX-M-1	1	7	(+)	N/A
	CTX-M-9	1	8:15	(+)	N/A
	Invalid test	1	-	(+)	N/A
*K. oxytoca* (*n* = 3)	CTX-M-1	3	6–7:15	(+)	N/A
*S. marcescens* (*n* = 2)	CXT-M-1	1	6:30	(+)	N/A
	Negative result	1	-	(+)	N/A
*C. freundii* (*n* = 2)	CTX-M-1	2	6–7	(+)	N/A
*P. mirabilis* (*n* = 1)	Invalid test	1	-	(+)	N/A

UI—uninterpretable-lack of any growth inhibition zone in the applied double disc synergy test (criteria described in the text); (+)—positive results in the applied method, N/A—not applicable.

**Table 4:** Comparison of the results obtained using Eazyplex^®^ SuperBug CRE assay and standard PCR for the chosen positive blood cultures-derived strains (*n* = 50).

Species Recovered from the Samples	Eazyplex^®^ SuperBug CRE Assay	No. of Isolates	Standard PCR	No. of Isolates
*K. pneumoniae* (*n* = 45)	CTX-M-1 CTX-M-1, CTX-M-9, NDM CTX-M-1, NDM Negative result Invalid test	39 1 3 1 1	CTX-M, TEM, SHV CTX-M CTX-M, TEM CTX-M, SHV CTX-M, NDM CTX-M, NDM CTX-M, TEM, SHV, NDM TEM CTX-M	22 8 7 2 1 2 1 1 1
*K. oxytoca* (*n* = 3)	CTX-M-1	3	CTX-M	3
*S. marcescens* (*n* = 2)	CXT-M-1 Negative result	1 1	CTX-M CTX-M, TEM	1 1

## Data Availability

The data presented in this study are not publicly available as a matter of confidentiality. However, these data are available upon request from the corresponding author.

## Supplementary Materials

The following are available online at https://repod.icm.edu.pl/dataverse/umk-medical-sciences as https://repod.icm.edu.pl/dataset.xhtml?persistentId=doi:10.18150/W6IXRP (accessed on 14 January 2002) Genie^®^II (OptiGene) User Manual (Instrument Software Version v2.22 (rc1).
